# Global Phylogeography of the Widely Introduced North West Pacific Ascidian *Styela clava*

**DOI:** 10.1371/journal.pone.0016755

**Published:** 2011-02-22

**Authors:** Sharyn J. Goldstien, Lise Dupont, Frédérique Viard, Paul J. Hallas, Teruaki Nishikawa, David R. Schiel, Neil J. Gemmell, John D. D. Bishop

**Affiliations:** 1 University of Canterbury, Christchurch, New Zealand; 2 Marine Biological Association, Plymouth, United Kingdom; 3 UMR 7618 BioEMCo, Equipe IBIOS, Université Paris-Est Creteil, Creteil, France; 4 UPMC Université Paris, Lab. AD2M, Station Biologique de Roscoff, Roscoff, France; 5 UMR 7144 CNRS UPMC, DivCo team, Station Biologique de Roscoff, Roscoff, France; 6 University of Glamorgan, Pontypridd, United Kingdom; 7 University of Plymouth, Plymouth, United Kingdom; 8 Toho University, Miyama, Chiba, Japan; 9 University of Otago, Dunedin, New Zealand; University of Cambridge, United Kingdom

## Abstract

The solitary ascidian *Styela clava* Herdman, 1882 is considered to be native to Japan, Korea, northern China and the Russian Federation in the NW Pacific, but it has spread globally over the last 80 years and is now established as an introduced species on the east and west coasts of North America, Europe, Australia and New Zealand. In eastern Canada it reaches sufficient density to be a serious pest to aquaculture concerns. We sequenced a fragment of the cytochrome oxidase subunit I mitochondrial gene (COI) from a total of 554 individuals to examine the genetic relationships of 20 *S. clava* populations sampled throughout the introduced and native ranges, in order to investigate invasive population characteristics. The data presented here show a moderate level of genetic diversity throughout the northern hemisphere. The southern hemisphere (particularly New Zealand) displays a greater amount of haplotype and nucleotide diversity in comparison. This species, like many other invasive species, shows a range of genetic diversities among introduced populations independent of the age of incursion. The successful establishment of this species appears to be associated with multiple incursions in many locations, while other locations appear to have experienced rapid expansion from a potentially small population with reduced genetic diversity. These contrasting patterns create difficulties when attempting to manage and mitigate a species that continues to spread among ports and marinas around the world.

## Introduction

An important aspect of biodiversity conservation and sustainability of marine resources is the mitigation of non-indigenous species (NIS). To-date, there have been no reported extinctions of native marine species caused by exotic invaders [1]. Nonetheless, they pose a serious threat to the sustainability of aquaculture concerns [2], [3] and they can alter the structure and composition of benthic communities [4], [5], thereby threatening global marine biodiversity and resource sustainability. With only 16% of the worlds' marine ecoregions free from NIS [6], invasive species are challenging pre- and post-border biosecurity strategies, and threatening biodiversity and ecosystem services around the world [7], [8]. However, invasions can also provide insight into community ecology dynamics [9], [10], competitive interactions [11] and the resilience of native assemblages [12] as NIS make their way into new ecosystems.

The combined availability of high-throughput molecular techniques and analyses of the resulting data based on explicit evolutionary models has caused a recent surge in the number of studies seeking to use genetic patterns to assess invasion pathways and the evolution of invasiveness [13], [14]. Recent reviews of these molecular studies show that a wide array of taxa, geographic scales, and molecular markers have been covered over the past decade [15], [16], with a range of results reported across both aquatic and terrestrial NIS [14]. A comprehensive analysis of molecular studies showed that conventional expectations of bottlenecks and reduced genetic variability for introduced populations do not always hold true for aquatic NIS, with only around 37% of studies reporting a significant loss of genetic variation in introduced populations [16]. However, most of these studies have sampled populations many years after the initial introductions and global spread. Marine taxa, also primarily sampled many years after introduction, exhibit a particularly wide range of molecular patterns. For example, high genetic diversity has been observed for the mitochondrial DNA gene cytochrome oxidase I (COI) in native and introduced populations of the ascidian *Microcosmus squamiger*. The first introduction of this species was recorded in1983 and it appears that extensive sharing of haplotypes has since occurred among populations [17]. In contrast, other ascidians, *Pyura praeputialis*, first recorded in 1985, and *Botryllus schlosseri,* first recorded in the 1830's, displayed genetic differentiation among native and introduced populations and between harbours and bays for this same gene [18], [19]. Other marine taxa also exhibit a considerable range of genetic diversities and differentiation for this gene. For example, native populations of the seaweed *Undaria pinnatifida*, first recorded outside of Asia in 1971, exhibit high genetic differentiation but low diversity, while introduced populations exhibit relatively high diversity [20], [21]. In contrast, the rapa whelk *Rapana venosa*, first recorded in 1940s, displayed high genetic diversity within its native range but was monomorphic throughout invaded Europe [22]. One of the clearer genetic patterns of introduction was seen for the Pacific acorn barnacle *Balanus glandula*. This species, recorded outside of North America since the 1960's, exhibits marked genetic structure within its native and invaded ranges, which enabled the identification of two different incursion pathways, one to Argentina and another to Japan [23]. Finally, the European green crab *Carcinus maenus*, a ubiquitous marine invader since its transport outside of Europe in the early 19^th^ century, has been comprehensively studied [24], [25], [26], [27], [28], [29]. In 1997, genetic identification confirmed cryptic invasions of this crab species [25], while two more recent studies [24], [29] found that genetic diversity was reduced in introduced populations, particularly in the newly invaded regions.

The solitary ascidian *Styela clava* Herdman, 1882 is one of many non-indigenous marine tunicates that now dominate fouling communities of ports and marinas around the world [30]. *S. clava* is native to the NW Pacific coastal regions of Japan, Korea, northern China and the Russian Federation. The first recorded introduction beyond this range was in California, probably in the 1920s (Lambert & Lambert 1998). In the Atlantic, this species was first observed in Britain, in Plymouth Sound and the adjacent Lynher River Estuary, in 1953 [31]. Established populations of *S. clava* are now recorded throughout the northern hemisphere, including Atlantic Europe plus a recent record from the Mediterranean basin [3], [32], [33], [34], Canada [2], [26], [35], both the eastern and western coasts of the USA [36], [37], [38] and several southern hemisphere harbours in Australia [39], [40] and New Zealand [41]. This successful invader occurs in a diverse array of habitats including man-made structures such as subtidal wharves, boat hulls and mussel ropes, as well as intertidal oyster racks. Large populations exist on intertidal rocky reefs and subtidal mud flats in native and some introduced locations. The diversity of habitats in which this species resides makes tracking and management of its spread particularly challenging.

Two recent studies of *Styela clava* used microsatellite markers and mtDNA to assess the role of human-mediated transport on regional expansion of this introduced species in England [42] and New Zealand [43]. These two locations respectively document one of the oldest and the newest reported incursions of *Styela clava*. Both studies suggested that extensive admixture was occurring through human-mediated transport within the regional locations. In addition, the New Zealand study showed that recreational vessels and commercial port vessels were both introducing new populations into New Zealand waters and recreational vessels were also responsible for post-border expansion of *S. clava* independent of the port populations [43]. Despite increased genetic differentiation suggesting multiple incursions, microsatellite allelic diversity in the younger New Zealand incursion was reduced in comparison to the older UK incursion. However, mtDNA haplotype diversity was high and the genetic distance among haplotypes was large [43] suggesting that the reduction in microsatellite allelic richness and heterozygosity is not necessarily due to founder effects, and may be better explained by admixture among divergent populations. A more recent study of *S. clava* using microsatellite markers also showed no evidence of reduced genetic diversity due to founder events throughout Europe, although evidence of population expansion and/or sub-structure was observed in many populations [44].

In this study of the solitary ascidian *Styela clava*, we assessed genetic diversity and its distribution among populations in an extensive global dataset of mitochondrial cytochrome oxidase I gene (COI) sequence. The long, well documented, chronology of global introductions makes this an ideal species to assess founder effects and changes in diversity with respect to time of introduction, as well as connectivity among populations. Based on the three recent molecular studies of this species, our expectation was that the mitochondrial diversity would not be reduced in newer incursions and that admixture among populations would be high for many populations due to their derivation from multiple sources. However, genetic differentiation among geographic regions was also expected, based on the differentiation of genotypes between the two native populations [44].

## Methods

A sub-set of partial fragments of the mitochondrial DNA oxidase subunit I mitochondrial gene (COI) for *Styela clava* from three different labs were aligned. Protocols for sample collection and DNA extraction can be found in Goldstien et al. [43] and Dupont et al. (2009). We reduced the effects of sampling errors with an extensive geographic sampling regime, including populations from all regions known to harbour this species. Unfortunately, we were only able to obtain samples from two populations within the native range, which limits our analysis to comparisons of diversity and assessing the connectivity among introduced populations. However, each sampled population is represented by 20–30 individuals, which provides a robust measure of diversity within, and structure among, populations. To avoid bias and oversampling in one location, only a subset of the 2006 data from Goldstien et al. (2010) were used; essentially, regional populations were excluded to avoid oversampling rare haplotypes.

### Sequence Analyses

All sequences were checked in the respective laboratories and were brought together for alignment. Alignments and haplotypes were identified and confirmed manually in Bioedit v. 5.0.6 [45]. Arlequin v. 3.0 [46] was used to calculate Nei's nucleotide diversity (π), computed as the probability that two randomly chosen homologous nucleotides are different (Nei, 1987), and theta(S), Watterson's theta: an estimate of the population mutation rate using the number of estimated from the infinite-site equilibrium relationship between the number of segregating sites, the sample size and θ (Watterson, 1975; Tajima, 1989). Haplotype number, as well as haplotypic richness and diversity contribution after rarefaction to a population size of 15, were estimated using CONTRIB [47]. A Statistical Parsimony Network was constructed in TCS 1.18 [48]. The divergence among haplotypes was calculated using the Kimura 2-parameter distance measure (Kimura, 1980) in MEGA4 [49]. To examine population structure independent of set population groupings and allowing for admixture, Bayesian Analysis of Population Structure (BAPS) was used in the program BAPS v.3.2 [50]. The hierarchical distribution of genetic variation among populations based on *a priori* population groupings was also tested using Analysis of Molecular Variance (AMOVA) [51] in Arlequin v. 3.0 [46] and was based on the number of pairwise nucleotide differences [52]. This simple distance measure was used due to the close genetic relationship of the sequences [53]. The populations were partitioned into regional groups: Japan, Europe, Australia, New Zealand, West USA, East USA, and Canada for this analysis. In addition, the genetic distance between populations was calculated using pairwise Φ_ST_ and Nei's average number of pairwise differences (Nei, 1979) in Arlequin v. 3.0 (Excoffier, 1992). Finally, to determine the relationship between ages since the introduction was reported and the genetic diversity for the populations; diversity measures Theta(S) and haplotypic richness, a Pearson's correlation test was done using XLstat (Addinsoft, 2006). A Non-linear regression was also performed in XLstat using the least squares method and default functions.

## Results

**Figure 1:**
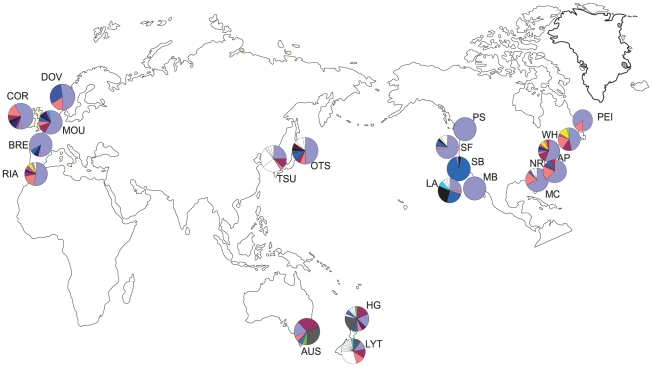
Haplotype distribution for the mtDNA COI gene of *Styela clava* populations sampled in 2006. Pie colours correspond to the haplotypes in Figure 2 and population codes follow Table 1.

A partial fragment, 602 base pairs, of the mitochondrial COI gene was aligned for a total of 554 *S. clava* individuals, derived from 20 populations around the world (Table 1, Fig. 1). Forty five haplotypes (GenBank accession numbers: GU328006-GU328035; HQ730795-HQ730809) were observed among the aligned sequence data from the 554 individuals sequenced and these haplotypes exhibited 0.2%–1.3% sequence divergence.

**Table 1:** Population locations, sample sizes (N) and summary statistics for *Styela clava*.

Population	Country	Sample ID	N	H	Pb15	Crt	Crs	Crd	U:S	π	Theta(S)
Otsuchi Bay	Japan	OTS	32	11	**5.99**	**0.014**	**0.023**	**0.000**	**3∶8**	**0.0024**	**3.48**
Tsukumo Bay	Japan	TSU	27	8	**5.19**	**0.064**	**0.015**	**0.049**	**5∶3**	**0.0052**	**3.11**
Prince Edward Isl.	Canada	PEI	19	2	0.99	0.000	0.000	0.000	0∶2	0.0005	0.29
Doverodde	Denmark	DOV	24	3	1.96	0.000	0.000	0.000	0∶3	0.0015	0.54
Brest	France	BRE	30	4	2.02	0.000	0.000	0.000	0∶4	0.0006	0.76
Cork	Ireland	COR	32	7	3.96	0.000	0.003	0.000	0∶7	0.0013	1.49
Ria de Ferrol	Spain	RIA	32	8	4.57	0.000	0.000	0.000	1∶7	0.0016	1.49
Plymouth	UK	MOU	30	8	4.66	0.000	0.010	0.000	0∶8	0.0013	1.77
Avery Point	East USA	AP	27	9	**5.12**	**0.003**	**0.014**	**0.000**	**0∶9**	**0.0016**	**2.08**
Mumford Cove	East USA	MC	22	5	0.52	0.000	0.000	0.000	2∶3	0.0010	1.10
Mission Bay	West USA	MB	31	1	0.00	0.000	0.000	0.002	0∶1	0.0000	0.00
Niwatec River	East USA	NR	22	5	3.27	0.000	0.000	0.000	0∶5	0.0015	1.32
Puget Sound	West USA	PS	24	1	0.00	0.000	0.000	0.000	0∶1	0.0000	0.00
Santa Barbara	West USA	SB	32	3	0.94	0.008	0.000	0.035	0∶3	0.0003	0.50
Los Angeles	West USA	LA	27	8	**5.03**	**0.036**	**0.013**	**0.023**	**3∶5**	**0.0022**	**1.82**
San Francisco	West USA	SF	33	6	3.07	0.000	0.000	0.000	2∶4	0.0004	1.23
Woods Hole	East USA	WH	30	6	3.89	0.000	0.002	0.000	1∶5	0.0016	1.26
Hauraki Gulf	New Zealand	HG	32	12	**7.21**	**0.050**	**0.035**	**0.015**	**4∶8**	**0.0042**	**3.48**
Lyttelton Hbr	New Zealand	LYT	31	13	**7.57**	**0.064**	**0.038**	**0.025**	**8∶5**	**0.0037**	**3.50**
Melbourne	Australia	AUS	16	6	4.81	0.036	0.011	0.025	0∶6	0.0035	1.81

The identification code for each population is included. Summary statistics are: H, number of haplotypes; Pb[15], haplotypic richness with rarefaction; Crt, contribution to total haplotypic richness; U:S, proportion of unique: shared haplotypes; π, nucleotide diversity; and Theta(S), the population mutation rate estimated from the number of segregating sites.

Note: Bold text highlights the populations with the highest diversity across multiple measures.

**Figure 2:**
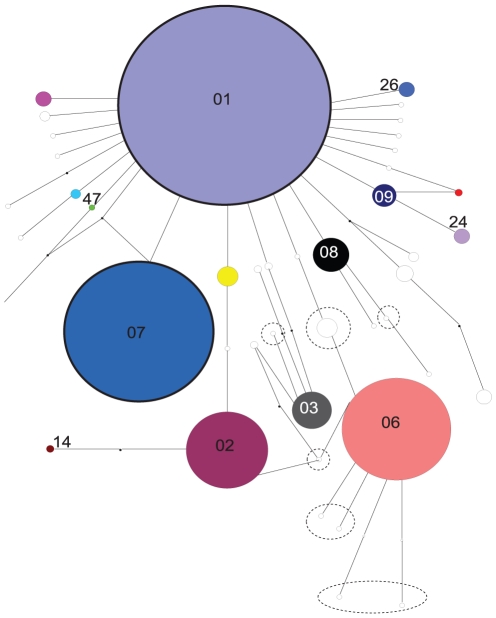
Statistical parsimony network for the mtDNA COI gene of *Styela clava* populations sampled in 2006. Unique haplotypes are not coloured and those unique haplotypes observed in the Lyttelton population are circled. Haplotypes that appear in the text are numbered accordingly.

**Figure 3:**
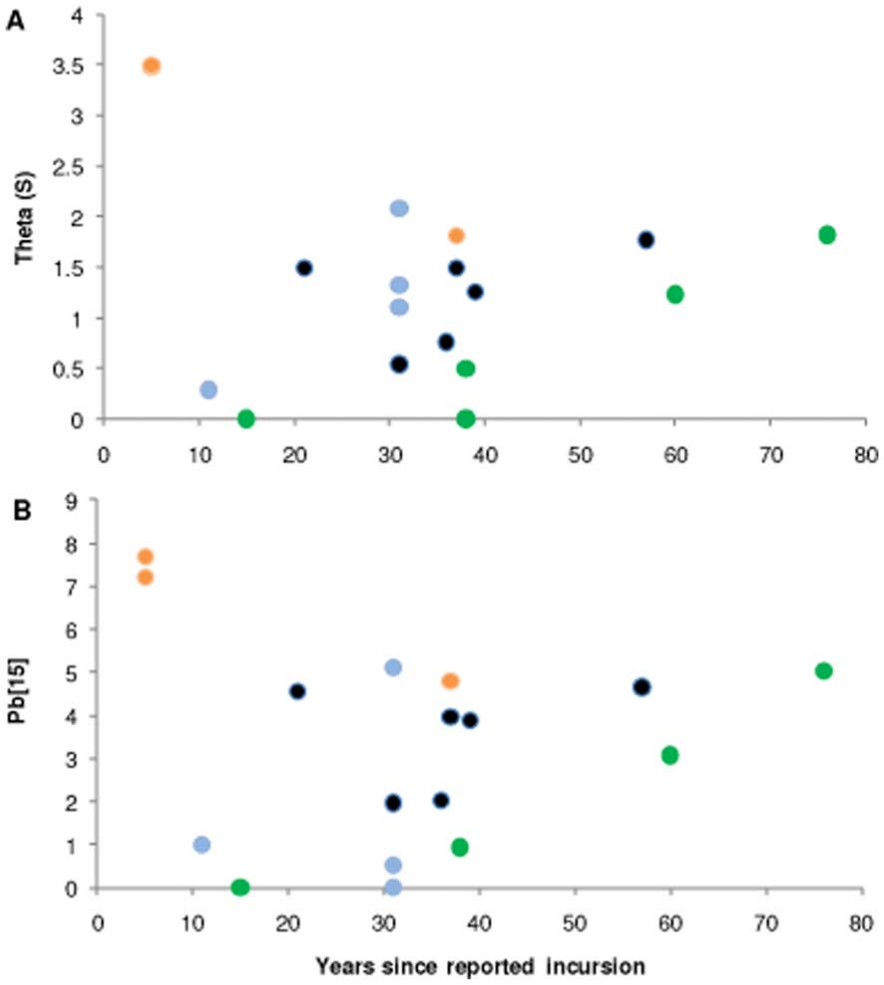
Scatter plots of theta(S) and haplotypic richness (Pb[**15**]), against years since the first reported incursion of *Styela clava* from 20 populations sampled globally (**Table 1**). Data points are coloured to represent populations from geographic regions: Europe (black), east coast Nth America (light grey), west coast USA (black stripe), and southern hemisphere (white).

The observed nucleotide diversity was low for almost all populations (π≤0.0052), while haplotype diversity was moderate, ranging between 1 and 13 haplotypes per population. New Zealand and Japan were the most genetically diverse populations and contributed most of the diversity to the total data set, as shown with π, Theta(S), and haplotypic richness (Table 1). The Lyttelton population was also consistent in its majority contribution to haplotypic and nucleotide diversity due to within population haplotype diversity (Crs) and haplotype divergence among populations (Crd), supported by the high number of unique haplotypes in this population, as well as nucleotide diversity and Theta(S) (Table 1). The remaining populations differed in contribution due to diversity or divergence of haplotypes, as well as nucleotide and θ_S_ diversity. The statistical parsimony network (Fig. 2) shows that unique haplotypes occurring in New Zealand, in particular, are more divergent than haplotypes in all other populations, contributing to the higher Theta(S) and Contribution (Crd,) values. Linear and non-linear equations did not show any significant correlation between age of incursion and the haplotypic richness (Pb15) or Theta(S) of the introduced populations, although an apparent positive trend is seen among the five populations from west coast USA (Fig. 3).

Two populations from the east (Otsuchi Bay) and west (Tsukumo Bay) coast of Honshu, mainland Japan, represent the native populations in this study, albeit a small area of the entire range. Both of these populations exhibit high genetic diversity (Table 1), and significant differentiation was observed between the two populations (Table S1). Eight of the eleven haplotypes from Otsuchi are spread throughout the introduced populations (Fig. 1; 01, 02, 03, 06, 07, 08, 09, 14), while only three of eight Tsukumo haplotypes are shared among other populations (Fig. 1; 01, 02, 06). One haplotype is shared among all populations (01), although this haplotype is less frequent in the southern hemisphere populations (Fig. 1). The frequency of each of the shared haplotypes differs among populations, particularly those in different ocean basins, such as between the west and east coast of the USA. For instance, the second most frequent haplotype (07) observed in west coast USA populations is rare in all other populations of the northern hemisphere, excluding Denmark, where it also occurs in high frequency. Similarly, the third most frequent haplotype (06) in Atlantic coast populations (i.e., Europe, east USA and east Canada), excluding France, occurs in very low frequency in west coast USA populations. Europe exhibits extensive admixture with generally low diversity; one unique haplotype is observed in Spain and one haplotype (24) is unique to the European countries of Spain (RIA), Ireland (COR) and France (BRE). Similarly, most of the North American populations exhibited low genetic diversity and extensive admixture. Los Angeles, San Francisco and Mumford Cove populations were the most diverse populations within the North American region.

The statistical parsimony network (Fig. 2) reflects the high frequency of some haplotypes and the large number of unique haplotypes observed among populations. There are also several missing, or hypothetical, haplotypes in the data most likely present in unsampled populations within the native range. The network also shows the wide range of divergence among haplotypes (0.2%–1.3%) with many of the unique haplotypes at the higher end of the divergence scale. Many of these divergent haplotypes occur in the Lyttelton population (Fig. 1). Genetic distance among populations (Table S1) shows that the two Japanese and two New Zealand populations are significantly different from most other populations. Lyttelton is the only population that is significantly different from all other populations. The Australian population is significantly different from all populations except the population of Huaraki Gulf, New Zealand. The southern hemisphere is genetically distinct from the northern hemisphere and from the native populations sampled. New Zealand populations exhibit a high number of unique haplotypes that are likely to be present in a native population not sampled. In particular, the Lyttelton population shares only five of its 13 haplotypes with other populations (one of these, 17, being shared only with the Los Angeles population), and two haplotypes (26 and 47) are unique to the Hauraki Gulf (Auckland, NZ) and Melbourne (Australia).

**Figure 4:**
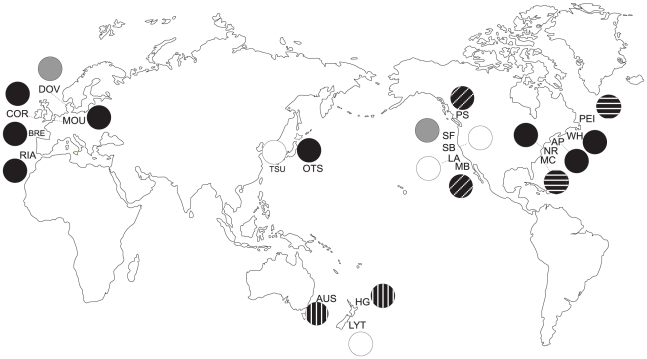
Bayesian population structure groups for the mtDNA COI gene of *Styela clava* determined using BAPS v.3.2. Circles are coloured to represent genetically similar populations. The unfilled circles represent populations that do not group with any others. Population codes follow Table 1.

BAPS groupings (Fig. 4) indicate genetic similarities among populations of the east coast of America and west coast of Europe, and Otsuchi; between Australia and Hauraki Gulf; between Prince Edward Island and Mumford Cove; between Doverode and San Francisco; and between Puget Sound and Mission Bay. Lyttelton, Tsukumo, Los Angeles and Santa Barbara did not group with any other populations. Analysis of Molecular Variance (AMOVA) was undertaken to quantify the components of genetic variance within the data (Table 2). Data were partitioned in two ways: 1) geographic location: Japan, Europe, west USA, east USA, east Canada, Australia, New Zealand, and 2) concordant with BAPS groupings (Figure 4). Both partitions show significant genetic structure among groups, but the degree of variation explained by the group was greater for BAPS partitions (Φ_CT_, 0.25) than for among geographic regions (Φ_CT_, 0.08). The variation within groups (Φ_SC_) is also reduced for the BAPS groups (Φ_SC_, 0.00) indicating that these groupings contain less variation than occurs within geographic regions (Φ_SC_, 0.15).

**Table 2:** F-statistics for *Styela clava* AMOVA results.

Source of variation	Among groups (Φ_CT_)	Among populations, within groups (Φ_SC_)	Within populations (Φ_ST_)
BAPS Groups	0.25	0.00	0.24
Geographical Regions	0.08	0.15	0.22

The data were partitioned in two ways: 1) BAPS Groups obtained from Bayesian analysis without prior population designation; 2) Populations grouped by geographical region. All results are significant (p<0.01).

Geographical regions: Japan, Europe, Australia, New Zealand, West USA, East USA, and Canada.

## Discussion

The aim of this study was to assess genetic diversity and its distribution among populations of *Styela clava* and to test the link between age of incursion and genetic diversity of this widely introduced ascidian. The COI gene revealed a moderate level of haplotype diversity (45 haplotypes in 554 individuals) with low to moderate nucleotide diversity (0.000–0.005), useful in identifying genetic similarities among populations. This level of haplotype diversity is similar to that observed for the star sea squirt, *Botryllus schlosseri*, for which 16 haplotypes were identified from 181 individuals throughout Europe [18]; however, the nucleotide diversity for this species was much higher (0.008 – 0.08). In contrast, two other ascidians exhibited high haplotype diversity across native and introduced populations: 52 COI haplotypes were observed in 258 individuals of an Australian ascidian, *Microcosmus squamiger*, now present in northern hemisphere locations [17], and 34 haplotypes were identified from 67 individuals of the Mediterranean ascidian *Cystodytes dellechiajei* [54]. *Microcosmus squamiger*, however, exhibited low nucleotide diversity (0.002 – 0.008) compared to the other ascidians, excluding *S. clava*, while for *Cystodytes dellechiajei* nucleotide diversity was also high (0.006 – 0.08) [17], [54].

Molecular studies have shown that genetic diversity of many introduced populations is equal to or greater than that of corresponding native populations, thereby contradicting theoretical expectations and possibly enhancing the success of invasive organisms [55], [56]. This observation is thought to result primarily through continuing introductions from multiple sources enhancing genetic diversity and increasing novel genotypes [14], [16]. The two native populations of *S. clava* sampled here exhibit significant genetic diversity and genetic differentiation from each other, and have high genetic diversity compared to most populations of the introduced range, with only New Zealand populations exhibiting similar genetic diversity (Table 1). The abundance of unique haplotypes in populations such as New Zealand and Los Angeles (Fig. 1) also suggests that a substantial component of the genetic variation in the native range of this species has not been sampled in this study. However, most of the haplotypes observed throughout the introduced range are observed in at least one of the native populations sampled.

Most of the haplotypes shared among northern hemisphere populations are also present in the Otsuchi Bay population, while haplotypes shared between Tsukumo Bay and introduced populations were also present in the Otsuchi Bay population (Fig. 1). The high frequency of unique haplotypes in Tsukumo Bay suggests that this population is not a likely source for any of the introduced populations sampled. Microsatellite data for Europe and USA populations showed a similar pattern of population clustering in the northern hemisphere [44]. As for this study, Tsukumo Bay was distinct from all other populations and links were suggested between Atlantic Europe and the eastern seaboard of the USA and between northern Denmark and the west coast of the USA, although for the microsatellite data it was the latter grouping that clustered with Otsuchi Bay. In Europe, neither the COI data presented here nor the microsatellite data of Dupont et al. (2010) shows a clear correlation between genetic diversity at a site and the time since *S. clava* was first reported there. In contrast, Los Angeles, which is very close to Newport Harbour where *S. clava* was first reported on the west coast of the USA (Abbott & Johnson 1972), did show higher COI diversity than other sites on this coast (Dupont et al. (2010) did not include this locality).

Molecular data for other marine invasive species of NE Asian origin have displayed weaker links with Japanese populations than were observed between introduced populations of *S. clava* and the Otsuchi Bay sample. For instance, native populations of the amphipod *Caprella mutica* in Japan were genetically diverse and all exhibited unique haplotypes that were not observed in any other locations in the data set [57]. Similarly, introduced populations of the brown seaweed *Undaria pinnatifida*, particularly in Europe but also in New Zealand and America, were genetically similar to aquaculture populations in Japan and Korea but less similar to natural Japanese populations [21].

The southern hemisphere *S. clava* populations are genetically distinct from the northern hemisphere introduced populations and from the native populations sampled, a pattern also observed for *U. pinnatifida* [20], [21]. New Zealand populations of *S. clava* exhibit a high number of unique haplotypes that are likely to be present in a native region not sampled. The significant genetic differentiation between the two New Zealand populations, Hauraki Gulf and Lyttelton, suggests that these ports received founders from different sources, most likely from vessels arriving from different locations. The same result was shown for microsatellite markers in New Zealand populations [43], but the more comprehensive data set presented here shows that Hauraki Gulf populations have stronger genetic affinities to Japan and the northern hemisphere populations than does the more southern port of Lyttelton.

It has been suggested that the build-up of genetic diversity from multiple sources is creating more successful invaders and that the founder effect may be overstated for NIS [14], [16], [58]. Our study does not support the idea that neutral genetic diversity is linked to invasive potential. Populations such as Prince Edward Island, Puget Sound and Mission Bay may well have received smaller incursions to account for their low diversity, but the invasiveness of these populations does not appear to be affected by their low genetic diversity. In particular, Prince Edward Island has experienced population numbers in pest proportions [59], [60], suggesting that the low founder diversity is not affecting the successful invasion of the species at this location. The species was also seasonally very abundant in Mission Bay during the surveys reported by Lambert & Lambert (1998). In addition, the high level of diversity within the Lyttelton population of New Zealand has not translated to high abundance, distribution, or competitive ability in this location (SJG unpublished data). Santa Barbara shows anomalously low diversity adjacent to a coastal region of high diversity (Los Angeles); in this case, chance events or selective processes may be acting within the enclosed environment of the marina to reduce genetic diversity subsequent to introduction.

One of the difficulties for biosecurity management of marine invasions is our ability to identify and date incursions. This species, like many others, shows a range of genetic diversities within populations and differentiation among them, independent of the age of incursion. Multiple incursions appear to be associated with the successful establishment of this species in many locations, while other locations have potentially experienced rapid expansion from a small founding population with reduced genetic diversity. The potential for multiple incursions blurs the line between founder events and the time required for genes to spread into established populations. These mixed patterns create difficulties when attempting to manage and mitigate a species that continues to spread.

## Supporting Information

Table S1 Pairwise comparisons for Φ_ST_ (above diagonal) and Nei's pairwise distance within populations (diagonal) and corrected distance among populations (below diagonal) of *S.clava* for mtDNA gene COI.
(DOC)
