# Supplementary material for: Global Phylogeography of the Widely Introduced North West Pacific Ascidian Styela clava
Source: PLoS One. 2011 Feb 22;6(2):e16755. doi: 10.1371/journal.pone.0016755 (PMC3043059; doi:10.1371/journal.pone.0016755)
Supplement: Table S1 — Pairwise comparisons for ΦST (above diagonal) and Nei's pairwise distance within populations (diagonal) and corrected distance among populations (below diagonal) of S.clava for mtDNA gene COI. (DOC) [file pone.0016755.s001.doc]

**Table S1.** Pairwise comparisons for Φ_ST_ (above diagonal) and Nei’s pairwise distance within populations (diagonal) and corrected distance among populations (below diagonal) of *S.clava* for mtDNA gene COI.

|  | **OTS** | **TSU** | **PEI** | **DOV** | **BRE** | **COR** | **RIA** | **MOU** | **AP** | **MC** | **MB** | **NR** | **PS** | **SB** | **LA** | **SF** | **WH** | **AKL** | **LYT** | **AUS** |
| --- | --- | --- | --- | --- | --- | --- | --- | --- | --- | --- | --- | --- | --- | --- | --- | --- | --- | --- | --- | --- |
| **OTS** | 1.44 | **0.18** | 0.01 | **0.04** | 0.02 | 0.01 | 0.02 | -0.01 | 0.01 | 0.01 | **0.04** | -0.01 | **0.03** | **0.47** | **0.08** | 0.02 | 0.02 | **0.12** | **0.18** | **0.13** |
| **TSU** | **0.48** | 3.15 | **0.22** | **0.25** | **0.26** | **0.22** | **0.22** | **0.23** | **0.22** | **0.22** | **0.30** | **0.21** | **0.26** | **0.49** | **0.26** | **0.27** | **0.23** | **0.20** | **0.24** | **0.19** |
| **PEI** | 0.02 | **0.57** | 0.28 | **0.15** | **0.07** | -0.01 | 0.00 | 0.01 | 0.04 | -0.03 | 0.16 | -0.02 | 0.13 | **0.80** | **0.16** | 0.04 | 0.01 | **0.14** | **0.19** | **0.20** |
| **DOV** | **0.06** | **0.68** | **0.09** | 0.69 | **0.13** | **0.12** | **0.11** | **0.08** | **0.11** | **0.13** | **0.28** | 0.05 | **0.25** | **0.48** | **0.08** | **0.09** | **0.13** | **0.18** | **0.24** | **0.25** |
| **BRE** | **0.02** | **0.57** | **0.03** | **0.08** | 0.38 | 0.03 | **0.06** | 0.00 | 0.01 | 0.07 | **0.05** | 0.04 | 0.03 | **0.73** | **0.15** | 0.02 | 0.10 | **0.21** | **0.30** | **0.30** |
| **COR** | 0.01 | **0.53** | 0.00 | **0.11** | 0.02 | 0.81 | -0.01 | -0.01 | 0.00 | 0.00 | **0.08** | -0.01 | **0.06** | **0.65** | **0.14** | **0.05** | 0.02 | **0.15** | **0.19** | **0.18** |
| **RIA** | 0.02 | **0.56** | 0.00 | **0.11** | **0.04** | -0.01 | 0.97 | 0.00 | 0.00 | 0.00 | **0.09** | -0.01 | **0.07** | **0.62** | **0.15** | **0.06** | -0.02 | **0.13** | **0.17** | **0.15** |
| **MOU** | -0.01 | 0.57 | 0.01 | 0.07 | **0.00** | -0.01 | 0.00 | 0.77 | -0.02 | 0.02 | **0.04** | -0.01 | 0.02 | **0.62** | **0.10** | 0.01 | 0.02 | **0.15** | **0.23** | **0.19** |
| **AP** | 0.01 | **0.57** | **0.03** | **0.11** | 0.01 | 0.00 | 0.00 | -0.01 | 0.98 | **0.05** | **0.06** | 0.01 | **0.05** | **0.61** | **0.13** | **0.04** | 0.01 | **0.15** | **0.23** | **0.18** |
| **MC** | 0.02 | **0.57** | -0.01 | **0.09** | **0.04** | 0.00 | 0.00 | 0.02 | **0.04** | 0.58 | **0.11** | -0.01 | **0.09** | **0.72** | **0.15** | **0.05** | 0.02 | **0.14** | **0.16** | **0.18** |
| **MB** | **0.03** | **0.61** | 0.02 | **0.11** | 0.01 | **0.03** | **0.05** | **0.01** | **0.03** | **0.03** | 0.00 | **0.10** | 0.00 | **0.90** | **0.22** | **0.04** | **0.12** | **0.24** | **0.33** | **0.37** |
| **NR** | 0.00 | **0.55** | -0.01 | 0.04 | **0.02** | -0.01 | -0.01 | -0.01 | 0.01 | -0.01 | **0.03** | 0.76 | **0.08** | **0.64** | **0.11** | 0.03 | 0.01 | **0.12** | **0.16** | **0.16** |
| **PS** | **0.03** | **0.61** | 0.02 | **0.11** | 0.01 | **0.03** | **0.05** | **0.01** | **0.03** | **0.03** | 0.00 | **0.03** | 0.00 | **0.89** | **0.19** | 0.03 | **0.10** | **0.21** | **0.30** | **0.32** |
| **SB** | **0.73** | **1.49** | **0.89** | **0.36** | **0.76** | **0.91** | **0.92** | **0.76** | **0.84** | **0.90** | **0.88** | **0.74** | **0.88** | 0.18 | **0.42** | **0.67** | **0.63** | **0.46** | **0.55** | **0.61** |
| **LA** | **0.12** | **0.77** | **0.18** | **0.09** | **0.15** | **0.18** | **0.21** | **0.12** | **0.18** | **0.19** | **0.18** | **0.14** | **0.18** | **0.51** | 1.35 | **0.12** | **0.16** | **0.20** | **0.26** | **0.24** |
| **SF** | 0.02 | **0.61** | 0.02 | **0.06** | 0.01 | **0.03** | **0.05** | 0.01 | **0.03** | **0.03** | **0.01** | 0.01 | 0.01 | **0.72** | **0.11** | 0.52 | **0.08** | **0.20** | **0.28** | **0.27** |
| **WH** | 0.03 | **0.60** | 0.02 | **0.12** | **0.07** | 0.02 | -0.02 | 0.02 | 0.01 | 0.02 | **0.06** | 0.01 | **0.06** | **0.94** | **0.22** | **0.06** | 0.94 | **0.12** | **0.16** | **0.14** |
| **AKL** | **0.26** | **0.69** | **0.31** | **0.39** | **0.41** | **0.29** | **0.26** | **0.31** | **0.32** | **0.30** | **0.41** | **0.27** | **0.41** | **1.17** | **0.50** | **0.39** | **0.25** | 2.55 | **0.08** | -0.04 |
| **LYT** | **0.41** | **0.83** | **0.36** | **0.50** | **0.57** | **0.36** | **0.32** | **0.45** | **0.49** | **0.32** | **0.56** | **0.33** | **0.56** | **1.44** | **0.66** | **0.53** | **0.31** | **0.20** | 2.23 | **0.07** |
| **AUS** | **0.24** | **0.66** | **0.27** | **0.39** | **0.38** | **0.25** | **0.22** | **0.28** | **0.28** | **0.26** | **0.38** | **0.24** | **0.38** | **1.25** | **0.52** | **0.36** | **0.20** | -0.09 | **0.17** | 2.13 |

Note: See Table 1 for location abbreviations. Bold text highlights significant differences between populations.
